# Glycogen Hydrogel Loaded with *Schistosoma japonicas* Peptide SJMHE1 Improves Skin Wound Healing

**DOI:** 10.3390/biom16030392

**Published:** 2026-03-05

**Authors:** Yanwei Yang, Shang Wang, Yuyun Jiang, Liyue Huo, Wei Zhu, Xiaolin Zhang, Yubei Zhang, Xuefeng Wang

**Affiliations:** 1 Department of Central Laboratory, The Affiliated Hospital of Jiangsu University, Zhenjiang 212001, China; 2 Tzu Chi International College of Traditional Chinese Medicine, Vancouver, BC V6H 1G7, Canada; 3 Department of Sports Medicine, The Affiliated Hospital of Jiangsu University, Zhenjiang 212001, China; 4 Department of Nuclear Medicine, Institute of Digestive Diseases, and Institute of Endocrinology, The Affiliated Hospital of Jiangsu University, Zhenjiang 212001, China

**Keywords:** SJMHE1 peptide, glycogen hydrogel, M2 macrophage polarization, angiogenesis, wound healing

## Abstract

Current wound healing strategies must confront numerous challenges. Helminth-induced immunomodulation offers a promising therapeutic avenue for inflammatory diseases and injury repair. However, research on the role of helminths in damage recovery remains limited. We utilized glycogen—a naturally occurring biomaterial—to encapsulate SJMHE1, a bioactive peptide derived from *Schistosoma japonicum*, and successfully developed a facilely prepared hydrogel formulation denoted as SJMHE1-gel. The properties of SJMHE1-gel, its effect on cell activity, and its performance in a murine full-thickness skin defect model were evaluated. The glycogen-based hydrogel exhibited a uniform pore size, excellent biocompatibility, and sustained release of SJMHE1. Topical application of SJMHE1-gel enhanced collagen deposition, promoted angiogenesis, facilitated the regeneration of hair follicles and sebaceous glands, and accelerated full-thickness wound healing. SJMHE1-gel also promoted M2 macrophage polarisation and suppressed inflammatory cytokine expression both in vivo and in vitro. Mechanistically, SJMHE1-treated macrophages upregulate TGF-β, which in turn promotes the migration of L929 fibroblasts and human umbilical vein endothelial cells (HUVECs) via the Smad3 pathway. Neutralization of TGF-β attenuates phosphorylated Smad3 (p-Smad3) levels and impairs the migratory capacity of both fibroblasts and HUVECs. Additionally, SJMHE1-treated macrophages upregulate VEGFA, thereby enhancing angiogenic tube formation in HUVECs. This easy-to-prepare hydrogel can regulate macrophage polarization, inhibit inflammation, promote angiogenesis, and accelerate collagen deposition, acting across wound healing stages to provide a novel therapeutic strategy.

## 1. Introduction

Skin damage repair is a dynamic and complex process involving the precise coordination of multiple cells and molecules to restore homeostasis following tissue injury [1]. This process includes hemostasis, inflammation, cell proliferation and migration, and tissue remodelling. Disruption or dysregulation of skin repair mechanisms can lead to the formation of non-healing wounds [2]. Furthermore, with an ageing population, non-healing wounds associated with conditions such as ageing, atherosclerosis, and diabetes represent a significant global public health challenge [3,4]. Current treatments for wound healing include a variety of gels, bioactive peptides, growth factors, and herbal preparations [5,6,7]. Despite extensive research, many strategies and advances in cutaneous wound healing, particularly for chronic, non-healing wounds, remain underdeveloped due to challenges in assessment and wound management. Therefore, the development of novel approaches to skin wound healing is of considerable medical importance worldwide.

Helminths have co-evolved with humans, developing sophisticated mechanisms to suppress the host immune system while promoting wound healing, tissue repair, and minimising pathological damage, thereby ensuring their survival within the host [8]. The therapeutic potential of helminth-induced immunomodulation for treating autoimmune and inflammatory diseases has been explored for many years, yielding promising results [8,9]. Moreover, helminth infections can reduce host tissue damage and stimulate tissue repair [8]. The liver fluke *Opisthorchis viverrini* (Mus musculus) secretes a granulin (GRN)-like growth factor, Ov-GRN-1, which promotes wound healing and angiogenesis [10]. Although recombinant Ov-GRN-1 is difficult to express, an easily synthesised bioactive peptide fragment of Ov-GRN-1 retains its wound healing-promoting properties in both in vivo and ex vivo models [11]. Migration of *Schistosoma japonicum* (*S. japonicum*) schistosomula can promote wound healing in mouse lungs [12]. *S. japonicum* soluble egg antigen (SEA) has been shown to inhibit insulin resistance and accelerate wound healing in a Lepr db/db diabetic mouse model [13]. Consequently, helminth-infected or helminth-derived products present a largely untapped source of therapeutic potential. Helminth-derived protein drugs could offer novel prospects for wound healing and the treatment of autoimmune diseases. Many nature-inspired drugs, such as venoms derived from various invertebrates and vertebrates, are commercially used to treat a range of diseases [14]. Helminth products have now been added to this growing pharmacopeia. Our previously characterised immunomodulatory peptide SJMHE1 from *S. japonicum* [15] inhibits delayed hypersensitivity (DTH) [16], collagenous arthritis (CIA) [17], asthma [18,19], acute and chronic colitis [20], allergic rhinitis [21], and psoriasis [22]. Moreover, SJMHE1 can promote the repair of peripheral nerve injuries in rats after sciatic nerve transaction [23]. However, peptide drugs are prone to instability and protease degradation during circulation.

Over the past decades, nanotherapeutics involving the encapsulation of various drugs and biomolecules in nanomaterials have shown superior therapeutic efficacy in enhancing wound healing [24,25,26]. Despite the availability of numerous nanomaterials, there is increasing interest in simple, cost-effective, non-toxic, biodegradable, and widely accessible natural nanoparticles. Glycogen, a non-toxic, biodegradable natural nanomaterial with a highly branched surface and dense core of nano-sized dendrimer-like structures, serves as a cellular energy source and is widely used in nanotherapeutics [27]. However, there is limited research on glycogen-based nanotherapy for wound healing.

Hydrogels are well-known for their moisturising properties and ability to absorb exudates, making them ideal for wound healing [28]. However, most multifunctional hydrogels involve complex preparation procedures. This complexity renders manufacturing processes difficult to control, which is unfavorable for large-scale production and hinders their clinical and commercial translation. In this study, a glycogen-based hydrogel loaded with SJMHE1 was developed for skin damage repair. The natural glycogen was modified by grafting with divinyltriamine (DETA) for amination, loaded with peptides, and subsequently mixed with polyvinyl alcohol (PVA) to produce a hydrogel containing SJMHE1 (SJMHE1-gel). The effect of SJMHE1-gel on skin wound healing in mice was evaluated, alongside its impact on fibroblast and HUVEC migration and macrophage polarisation in vitro. The SJMHE1-encapsulated glycogen hydrogel accelerates skin wound healing in mice by alleviating inflammation, fostering angiogenesis, and boosting collagen deposition. Notably, it engages in all key phases of the murine wound healing process—encompassing the inflammatory, proliferative, and remodeling stages. As such, this hydrogel emerges as a multifunctional option characterized by its simplicity and ease of preparation.

## 2. Materials and Methods

### 2.1. Preparation of SJMHE1-Gel

The SJMHE1 peptide was purchased from SynPeptide Co., Ltd. (Nanjing, China), which had a purity exceeding 98%, as determined by high-performance liquid chromatography (HPLC). AG was synthesised according to a previously reported method [29]. The SJMHE1-gel was prepared as follows: First, an 18% (wt%) PVA solution was mixed with glycerol and PEG 400 in a 10:1:1 (*v*/*v*/*v*) ratio. The mixture was stirred thoroughly to form the PVA hydrogel. Next, the AG solution was combined with the peptide solution at a weight ratio of 2:1 (AG/peptide) and incubated on ice for 1 h. The resulting AG-peptide complex was then added to the PVA hydrogel, achieving a final peptide concentration of approximately 0.2 mg/mL. The prepared SJMHE1-gel was sealed and stored at 4 °C.

### 2.2. Release of Peptide from the SJMHE1-Gel

To prepare FITC-labelled SJMHE1 hydrogel, the above method was followed. The FITC-SJMHE1-gel (2 mL) was placed into a dialysis bag (3.5 kDa) and then submerged in 50 mL of water in a centrifuge tube. At each time interval, 200 μL of water was withdrawn from the tube to measure the fluorescence intensity using a SpectraMax M2 microplate reader (Molecular Devices, San Jose, CA, USA). The concentration of released peptide was calculated by interpolating the fluorescence values from a standard curve generated with serial dilutions of FITC-SJMHE1. Fresh water (200 μL) was added to the tube after each sample withdrawal.

### 2.3. Scanning Electron Microscopy (SEM) Analysis

Hydrogel samples were subjected to freeze-drying using a freeze-drier (Telstar-85 plus, Telstar, Barcelona, Spain), embrittled in liquid nitrogen, and a gold-palladium layer was applied to the cross-section. The morphology of the hydrogel slices was examined using a scanning electron microscope (ZEISS Sigma 360, ZEISS, Oberkochen, Germany) at an accelerating voltage of 3.0 kV.

### 2.4. Cell Viability Assay

To assess cytotoxicity, 0.2 g gel or SJMHE1-gel (containing 40 μg SJMHE1 at a concentration of 0.2 mg/mL) was added to 1 mL of complete medium and incubated at 37 °C for 24 h to prepare the hydrogel extract [30]. The extract was filtered through a 0.22 μm membrane filter. Mouse fibroblasts (L929) were seeded into 96-well plates at a density of 5 × 10^3^ cells per well and incubated for 24 h to allow for adhesion. Following attachment, the hydrogel extract was added to the culture wells and incubated for a further 24 or 48 h. Cell viability was measured using CCK-8 (C0038, Beyotime, Shanghai, China), following the manufacturer’s instructions.

### 2.5. Hemocompatibility Assessment

To evaluate haemolysis, whole mouse blood was centrifuged at 1500× *g* for 10 min to isolate plasma. Erythrocytes were washed twice with PBS and resuspended in PBS to create a 5% (*v*/*v*) solution. Aliquots of 900 μL of the erythrocyte solution were mixed with 100 μL of hydrogel containing SJMHE1 at a concentration of 0.2 mg/mL, gel, 0.1% Triton X-100 (positive control), or PBS (negative control). After incubation at 37 °C for 2 h, the mixtures were centrifuged at 1500× *g* for 10 min. The absorbance of the supernatant was measured at 540 nm using a microplate reader.

### 2.6. Skin Injury Induction and Topical Treatment

Female C57BL/6 mice (6–8 weeks old) were obtained from the Animal Experiment Centre of Jiangsu University. After anaesthesia with 2% pentobarbital sodium, the dorsal fur of the mice was shaved, and depilatory cream was applied to remove any remaining hair. The skin was then cleaned with alcohol-soaked cotton balls. A full-thickness wound, 8 mm in diameter, was created on the back using a disposable sterile biopsy punch [31]. Following wound induction, the mice were randomly assigned to four groups (*n* = 12 per group): the model group (no treatment), the gel group (treated with 50 μL of blank hydrogel), the SJMHE1-gel group (treated with 50 μL of SJMHE1-gel at a concentration of 0.2 mg/mL), and collagenase group (positive control, treated with 50 μL of collagenase ointment; Crand Life Sciences (Anshan) Co., Ltd., Anshan, China). Except for the model group, the wounds in each group were treated topically with the corresponding preparation once daily. All animals received humane treatment and were housed according to the Guidelines for the Protection and Use of Experimental Animals and the Measures for the Administration of Animal Use at Jiangsu University. All animal procedures were approved by the Institutional Animal Care and Use Committee (IACUC) of Jiangsu University (Permit Number: UJS-IACUC-AP-2024032016).

### 2.7. Wound Closure Analysis

Wounds were photographed on days 0, 3, 7, and 11 post-injury. Wound size was quantified using ImageJ software (version 1.54g, National Institutes of Health, Bethesda, MD, USA; https://imagej.nih.gov/ij/, access on 1 February 2026), and the wound healing rate was calculated using the formula: [(Wound Area (Day 0) − Wound Area (Day N))/Wound Area (Day 0)] × 100%. Mice were euthanized on days 7 and 11 (*n* = 6 per group at each time point) by sodium pentobarbital overdose (150 mg/kg, i.p.) followed by cervical dislocation. Skin surrounding the wound site (approximately 1 cm in diameter) was excised. A portion of the excised skin was snap-frozen in liquid nitrogen and stored at −80 °C; the remaining portion was fixed in 4% paraformaldehyde for subsequent histological examination.

### 2.8. Histological Analysis

Wound tissue fixed in 4% paraformaldehyde was paraffin-embedded and sectioned at a thickness of 5 µm using a microtome. Following deparaffinisation, sections were stained. Haematoxylin and eosin (H&E) staining was used to assess inflammatory cell infiltration, wound length, and epidermal thickness. Specifically, inflammatory cell infiltration was assessed by counting the number of inflammatory cells per high-power field (HPF) [18]. Wound length was defined as the distance between the two wound margins within the epidermal layer. Epidermal thickness was quantified by randomly selecting five points within the wound area using ImageJ software, and the mean value was calculated for each sample [32]. Collagen fibre deposition was evaluated using Masson’s trichrome staining.

### 2.9. Immunohistochemistry and Immunofluorescence

For antigen retrieval, paraffin-embedded mouse wound tissue sections were deparaffinised and hydrated. Sections were incubated overnight at 4 °C with primary antibodies: anti-collagen I (GB11022, Servicebio, Wuhan, China; diluted 1:200), anti-CD31 (GB11063-3, Servicebio; diluted 1:100), anti-TGF-β1 (sc-52893, Santa Cruz, CA, USA; diluted 1:50), anti-VEGFA (GB15165, Servicebio; diluted 1:200), and anti-p-Smad3 (AP1263, ABclonal, Wuhan, China; diluted 1:100). After primary antibody incubation, sections were treated with horseradish peroxidase (HRP)-conjugated secondary antibody (GB23303, GB23301, Servicebio; diluted 1:200) for 1 h at room temperature. Immunoreactivity was visualised using DAB chromogen, followed by counterstaining with haematoxylin. Images were captured using a microscope (Olympus CH51, Tokyo, Japan). Quantitative analysis was performed using ImageJ software. The expression levels of collagen I, TGF-β1, VEGFA, and p-Smad3 were assessed by measuring the average optical density (AOD) of positively stained areas [33]. Additionally, the number of CD31-positive microvessels was counted in three randomly selected fields per section [34].

For tissue immunofluorescence staining, sections underwent the same initial treatments as for immunohistochemistry. Sections were then incubated overnight at 4 °C with primary antibodies: anti-F4/80 (GB11027, Servicebio; diluted 1:500), anti-iNOS (GB11119, Servicebio; diluted 1:200), and anti-Arg1 (GB11285, Servicebio; diluted 1:200). After washing with PBS, sections were incubated for 1 h with the corresponding fluorescently labelled secondary antibodies (GB25303, GB21303, Servicebio, Wuhan; diluted 1:400) in the dark. Nuclei were counterstained with DAPI for 5 min. Images were acquired using a fluorescence microscope (Olympus BX51, Tokyo, Japan). For quantitative analysis, ImageJ software was used to count the numbers of F4/80^+^iNOS^+^ (M1) and F4/80^+^Arg1^+^ (M2) macrophages, and the proportions of iNOS^+^ M1 and Arg1^+^ M2 macrophages relative to the total F4/80^+^ macrophage population were calculated.

### 2.10. SJMHE1-Gel Tracking in Wound Tissue

To assess SJMHE1 peptide retention and distribution in wound tissue, a separate cohort of mice (*n* = 6) were subjected to wound creation and treated with FITC-labelled SJMHE1 hydrogel as described above. On day 5 following treatment, mice were euthanized by sodium pentobarbital overdose (150 mg/kg, i.p.) followed by cervical dislocation. The wound tissues were collected and immediately frozen at −80 °C. For immunofluorescence, sections were incubated overnight at 4 °C with the primary antibody anti-F4/80 (GB11027, Servicebio; diluted 1:500). After washing with PBS, sections were incubated with fluorescently labelled secondary antibodies (GB21303, Servicebio; diluted 1:400) for 1 h at room temperature in the dark. Nuclei were counterstained with DAPI for 5 min. Images were acquired using a fluorescence microscope (Olympus BX51, Tokyo, Japan).

### 2.11. Cell Culture and Treatment Procedures

RAW264.7, L929, and THP-1 cells were obtained from Shanghai Zhong Qiao Xin Zhou Biotechnology (Shanghai, China). RAW264.7 and L929 cells were cultured in DMEM medium supplemented with 10% fetal bovine serum (FBS), while THP-1 cells were cultured in suspension in RPMI-1640 medium with 10% FBS. HUVECs were purchased from Fenghui Biotechnology (Wuhan, China) and maintained in endothelial cell-specific medium. All cells were routinely cultured at 37 °C with 5% CO_2_ under saturated humidity, and cells in the logarithmic growth phase were used for experiments.

RAW264.7 cells were seeded into 6-well plates and cultured at 37 °C with 5% CO_2_. The cells were divided into three groups: control, LPS (L2880, Sigma-Aldrich, St. Louis, MO, USA), and LPS + SJMHE1. For the LPS + SJMHE1 group, cells were pretreated with 1 μg/mL SJMHE1 for 3 h, followed by exposure to 1 µg/mL LPS for 24 h.

### 2.12. Flow Cytometry

RAW264.7 cells were collected, centrifuged, and the supernatant discarded. The cells were then resuspended in staining buffer with anti-CD11b antibody (101206, Biolegend, San Diego, CA, USA) and incubated for 30 min at 4 °C. After washing twice, 250 μL of fixative (554655, BD Biosciences, San Diego, CA, USA) was added, and the cells were fixed for 20 min. Subsequently, cells were resuspended in 1× wash buffer (554723, BD Biosciences) with anti-CD206 antibody (141706, Biolegend) and incubated for 30 min at 4 °C. After two washes, the cells were resuspended in staining buffer and analysed using flow cytometry.

### 2.13. Quantitative Real-Time PCR

Total RNA was isolated from skin tissues and cells using VeZol Reagent (R411-01, Vazyme, Nanjing, China). cDNA was synthesised using a Reverse Transcription Kit (R211-01, Vazyme) according to the manufacturer’s instructions. Quantitative real-time PCR (qRT-PCR) was performed using the ChamQ Universal SYBR qPCR Master Mix (Q711-02, Vazyme) on the QuantStudio 5 Real-Time PCR System (Thermo Fisher Scientific, Waltham, MA, USA). Primers were obtained from Tsingke Biotech Co., Ltd. (Nanjing, China). The expression levels of target genes were quantified using the 2^−ΔΔ^Ct method, with results normalised to GAPDH expression levels.

### 2.14. Western Blotting

Cells were lysed using RIPA buffer supplemented with protease inhibitors. Protein samples were separated by 10% sodium dodecyl sulfate-polyacrylamide gel electrophoresis (SDS-PAGE) and subsequently transferred to polyvinylidene difluoride (PVDF) membranes. After transfer, the membranes were blocked and incubated overnight at 4 °C with primary antibodies: anti-β-actin (AF7018, Affinity, Jiangsu, China; diluted 1:5000), anti-GAPDH (10494-1-AP, Proteintech, Wuhan, China; diluted 1:10,000), anti-Arg1 (16001-1-AP, Proteintech; diluted 1:5000), anti-TGF-β1 (Santa Cruz; diluted 1:500), anti-VEGFA (A0280, ABclonal; diluted 1:1000), and anti-p-Smad3 (AP1263, ABclonal; diluted 1:1000). After washing with TBST, membranes were incubated with horseradish peroxidase (HRP)-conjugated secondary antibodies (RGAR001, RGAM001, Proteintech; diluted 1:5000) for 1 h at room temperature, followed by additional TBST washes. Bands were visualised using the ECL Chemiluminescent Substrate Kit (BMU102-CN, Abbkine, Wuhan, China) and captured with a chemiluminescence imager (Tanon 5200, Shanghai, China). Quantification was performed using ImageJ software.

### 2.15. Preparation of Conditioned Medium

THP-1 cells were treated with 100 ng/mL PMA (S1819, Beyotime) for 24 h to induce differentiation into macrophages. RAW264.7 cells and PMA-differentiated THP-1 macrophages were cultured in complete medium with or without 1 μg/mL SJMHE1 for 24 h. The supernatant was collected, filtered through a 0.22-μm filter, and then mixed with fresh medium at a 1: 2 (*v*/*v*) ratio to obtain macrophage-conditioned medium (CM-control and CM-SJMHE1). The CM was stored at 4 °C for later use.

### 2.16. Scratch Assay

A scratch assay was performed using L929 cells and HUVECs. Cells were seeded in 6-well plates and grown to 80% confluence. A straight scratch was introduced into the confluent monolayer using a sterile pipette tip. After washing with PBS, L929 cells and HUVECs were treated with the following low-serum (2% FBS) conditioned media: CM-Control, CM-SJMHE1, CM-SJMHE1 supplemented with 5 μg/mL TGF-β1 neutralising antibody (BE0057, Bio X Cell, Lebanon, PA, USA), or normal medium supplemented with 10 ng/mL TGF-β1 recombinant protein (HY-P70648, MedChemExpress, Monmouth Junction, NJ, USA). HUVECs were treated with CM from THP-1-induced macrophages. The cells were cultured at 37 °C in a 5% CO_2_ incubator. Images of the scratch wound were captured at fixed positions at 0, 24, and 48 h post-scratching. Cell migration was quantified by counting the number of cells that had migrated into the scratch area using ImageJ software.

### 2.17. Tube Formation Assay

Matrixgel was thawed overnight at 4 °C, and 50 μL of matrixgel was added to each well of a 96-well plate, and then incubated at 37 °C for 45 min. HUVECs were collected and resuspended in CM. A total of 1 × 10^4^ cells were seeded in each well and incubated at 37 °C with 5% CO_2_ for 4 h. Cell tube formation was photographed using an inverted microscope and analysed statistically using ImageJ software.

### 2.18. Statistical Analysis

Statistical analyses were performed using GraphPad Prism software (version 9.5.0). Data are presented as mean ± standard deviation (SD). Comparisons between two groups were analysed using unpaired Student’s *t*-tests. Comparisons among three or more groups were conducted using one-way analysis of variance (ANOVA) followed by Tukey’s multiple comparisons test. Statistical significance was defined as *p* < 0.05.

## 3. Results

### 3.1. Characterisation and Biocompatibility of the Hydrogels

**Figure 1:**
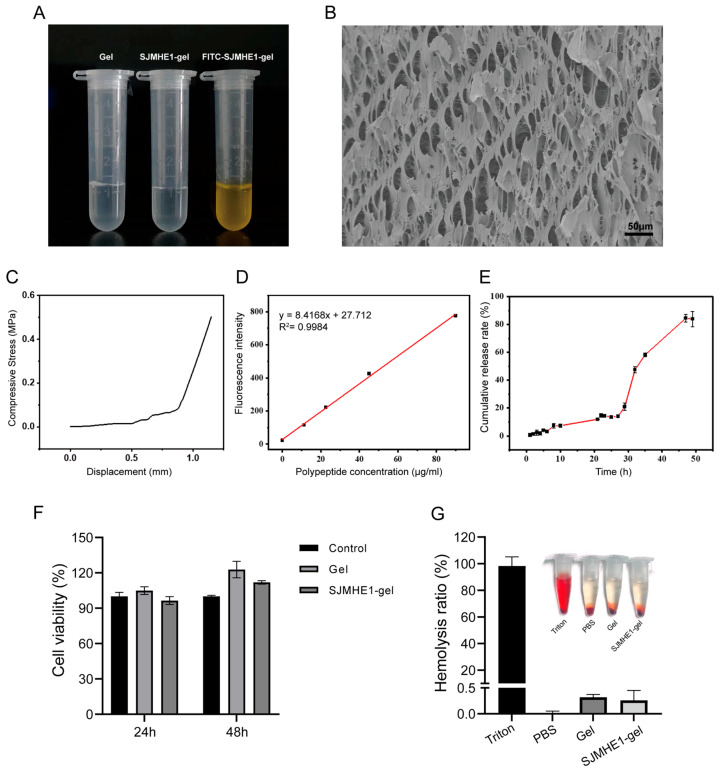
Characterisation and biocompatibility of hydrogels. (**A**) Hydrogel images. (**B**) SEM image of hydrogels (scale bar = 50 μm). (**C**) Compressive stress of hydrogels. (**D**) Standard curve correlating peptide concentration with fluorescence intensity. (**E**) SJMHE1 release profile. (**F**) Cytotoxicity of hydrogels. (**G**) Hemolysis rate of hydrogel.

A simple and cost-effective method for preparing glycogen-loaded SJMHE1 hydrogel was developed by mixing AG with SJMHE1 peptide and PVA gel, resulting in the hydrogel termed SJMHE1-gel. The appearance and morphology of the hydrogel are shown in Figure 1A and Supplementary Figure S1, with the FITC-labelled SJMHE1 peptide uniformly distributed throughout the matrix. Scanning electron microscopy was used to examine the hydrogel’s microstructure, revealing a network of interconnected, uniformly distributed pores (Figure 1B). This porous structure facilitates drug loading. Mechanical testing revealed that the hydrogel exhibited a maximum compressive stress of approximately 0.5 MPa (Figure 1C), indicating its elasticity and tensile strength.

FITC-labelled SJMHE1 was used to prepare glycogen-loaded peptide hydrogels, and the peptide release profile was subsequently evaluated (Figure 1D). The cumulative release of SJMHE1 peptide from the hydrogels reached ~85% at 48 h (Figure 1E), characterized by an initial slow-release period (0–28 h, ~20%) followed by a rapid-release period (28–48 h, ~80%), and ultimately reaching a plateau (~85% cumulative release at 48–50 h).

Fibroblasts play a central role in collagen synthesis, matrix remodelling, and contraction during wound healing [35,36], with their proliferation serving as a key indicator of material biocompatibility. The cytotoxicity of the SJMHE1 hydrogel was assessed using the CCK-8 assay, with the L929 fibroblast cell line co-incubated with the hydrogel extract for 24 or 48 h. As shown in Figure 1F, the hydrogel extracts did not inhibit cell proliferation, demonstrating excellent cytocompatibility. The hemocompatibility of the SJMHE1 hydrogel was evaluated through an in vitro hemolysis assay. The results, shown in Figure 1G, indicate that the hemolysis rate for both the blank and SJMHE1 hydrogels was below 5%, confirming their excellent hemocompatibility.

### 3.2. SJMHE1-Gel Treatment Promotes Wound Healing in Mice

**Figure 2:**
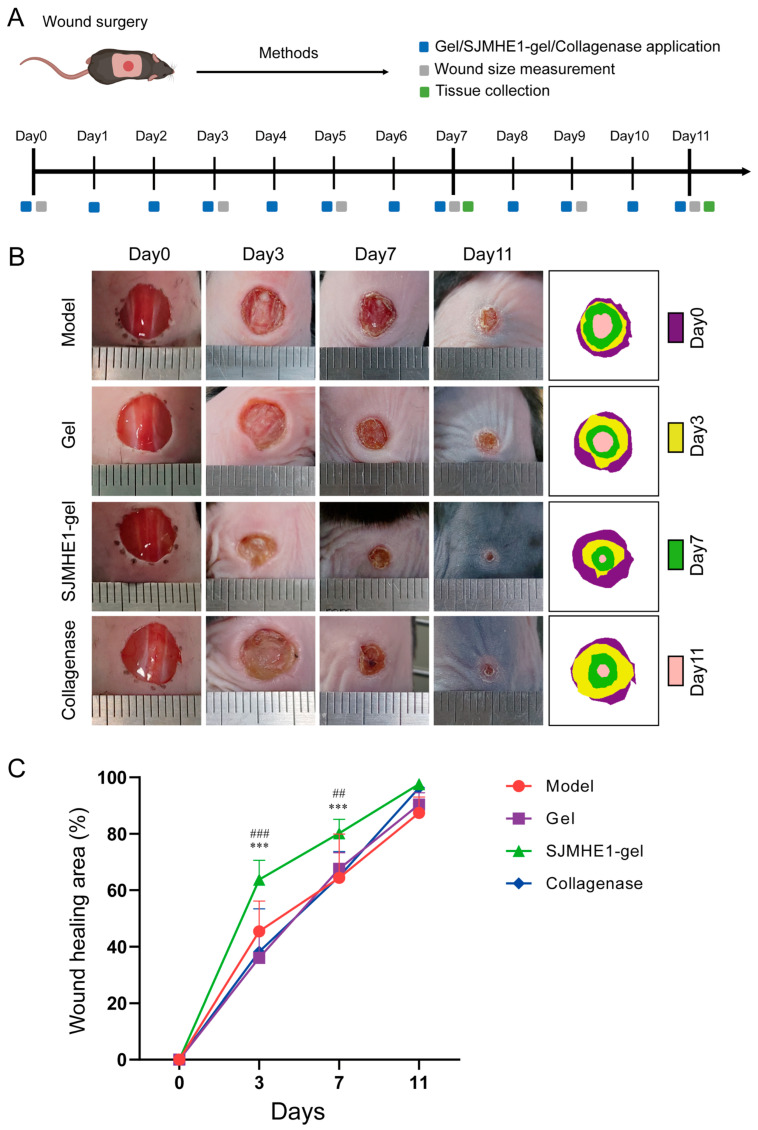
Visual assessment of wound healing. (**A**) Experimental protocol for treating skin lesions with hydrogel. (**B**) Photographs of wound healing progression in mice on various treatment days. (**C**) Wound healing rate graph. Data are presented as mean ± SD (*n* = 6; *** *p* < 0.001 for SJMHE1-gel group vs. model group, ## *p* < 0.01 and ### *p* < 0.001 for SJMHE1-gel group vs. gel group).

To assess the effect of SJMHE1-gel on wound healing in mouse skin, a dorsal full-thickness wound model was established. The experimental procedure is outlined in Figure 2A. The wound healing process was observed on days 0, 3, 7, and 11. Representative images are shown in Figure 2B, while the quantitative healing rates are presented in Figure 2C. The results revealed that the SJMHE1-gel group exhibited a significantly accelerated healing rate compared to the other groups. On day 3, the healed area in the SJMHE1-gel group reached 63.7 ± 6.9%, notably higher than that in the model group (45.4 ± 10.7%), the gel group (36.0 ± 9.9%), and the collagenase group (38.2 ± 15.2%). By day 7, the healed area in the SJMHE1-gel group (80.2 ± 5.0%) remained significantly greater than in the model group (64.4 ± 15.5%), the gel group (67.5 ± 5.8%), and the collagenase group (64.9 ± 8.9%). On day 11, wounds in the SJMHE1-gel group (97.6% ± 1.7%) and the collagenase group (96.3% ± 1.2%) were nearly fully healed, while the model group (87.4% ± 5.6%) and the gel group (90.4% ± 4.2%) still exhibited partial scabbing, indicating incomplete healing. These results suggest that SJMHE1-gel treatment significantly enhanced skin wound healing in mice, outperforming the positive control, collagenase, in the early stages of wound repair.

### 3.3. SJMHE1-Gel Treatment Reduces Inflammation and Promotes Wound Reorganisation

**Figure 3:**
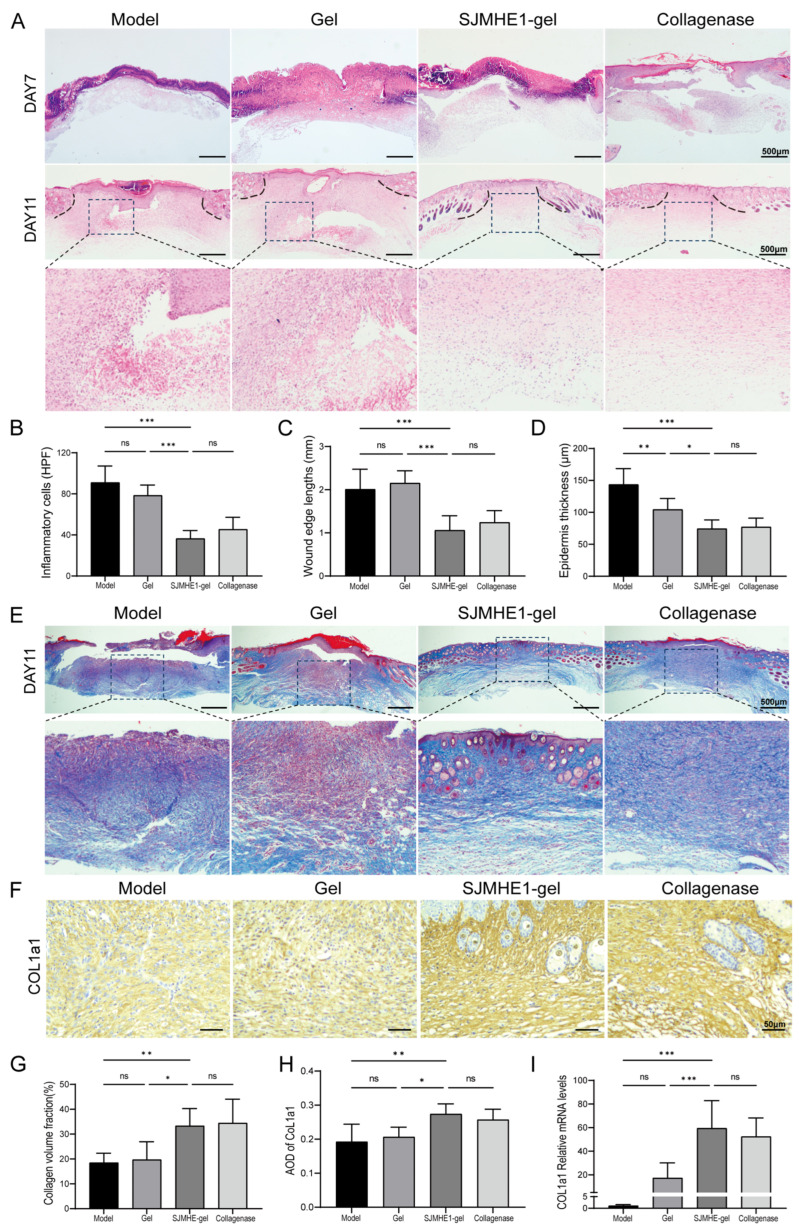
SJMHE1-gel accelerates wound healing in mice. (**A**) H&E-stained wound sections on postoperative days 7 and 11 (scale bar = 500 μm). (**B**) Inflammatory cell count on day 11. (**C**) Wound edge lengths on day 11 for each group. (**D**) Epidermal thickness in each group on day 11. (**E**) Masson’s trichrome staining of wound sections on postoperative day 11 (scale bar = 500 μm). (**F**) Representative images of immunohistochemical staining of CoL1a1 on day 11. (**G**) Quantification of collagen volume fraction from Masson’s staining on day 11. (**H**) Quantification of Col1a1 expression. (**I**) CoL1a1 gene expression levels on day 11. Data are presented as mean ± SD (*n* = 6). ns = not significant, * *p* < 0.05, ** *p* < 0.01, *** *p* < 0.001.

To further investigate the wound-healing effects of SJMHE1-gel, histological analysis was performed to examine the pathological changes in skin tissues during the healing process. Figure 3A shows the HE staining images of wound tissues on days 7 and 11. On day 7, neoepithelial tissue began to form at the wound edges in the SJMHE1-gel group. On day 11, the final stage of wound healing, mice treated with SJMHE1-gel or collagenase achieved complete re-epithelialisation. The SJMHE1-gel group (36.5 ± 7.8 cells/HPF) exhibited significantly reduced inflammatory cell infiltration compared to the model group (91.2 ± 16.0 cells/HPF) (Figure 3B) and significantly shorter wound lengths compared to both the model and gel groups (Figure 3C). Additionally, the epidermal thickness in the SJMHE1-gel group was significantly thinner than that in the model and gel groups, approaching that of normal skin (Figure 3D).

On day 11, collagen fibres in the model and gel groups were sparsely distributed and disorganised. In contrast, the SJMHE1-gel group exhibited more densely packed and orderly arranged collagen fibres, forming a wavy or reticular structure (Figure 3E). The collagen deposition in the SJMHE1-gel group was significantly higher than that in the model and gel groups (*p* < 0.01), with no significant difference observed compared to the collagenase group (Figure 3G). COL1a1, a major collagen component, serves as a marker of tissue repair during wound healing [37]. Immunohistochemical staining for COL1a1 was evaluated by semi-quantitative analysis of the average optical density (AOD). The results confirmed that COL1a1 expression in the wound tissues of the model and gel groups was relatively low (model: 0.19 ± 0.05; gel: 0.21 ± 0.03). In contrast, the SJMHE1-gel group exhibited a significantly higher COL1a1 expression level (0.27 ± 0.03) (Figure 3H).

qRT-PCR analysis confirmed that COL1a1 mRNA expression was significantly elevated in the wound tissues of mice treated with SJMHE1-gel compared to those in the model and gel groups (*p* < 0.001), with no significant difference observed compared to the collagenase group (Figure 3I). These results collectively demonstrate that SJMHE1-gel treatment reduced inflammation, accelerated neoepithelial tissue formation, promoted collagen synthesis and deposition, and significantly improved the tissue structure of healed wounds.

### 3.4. SJMHE1-Gel Treatment Promotes Angiogenesis During Early Wound Healing

**Figure 4:**
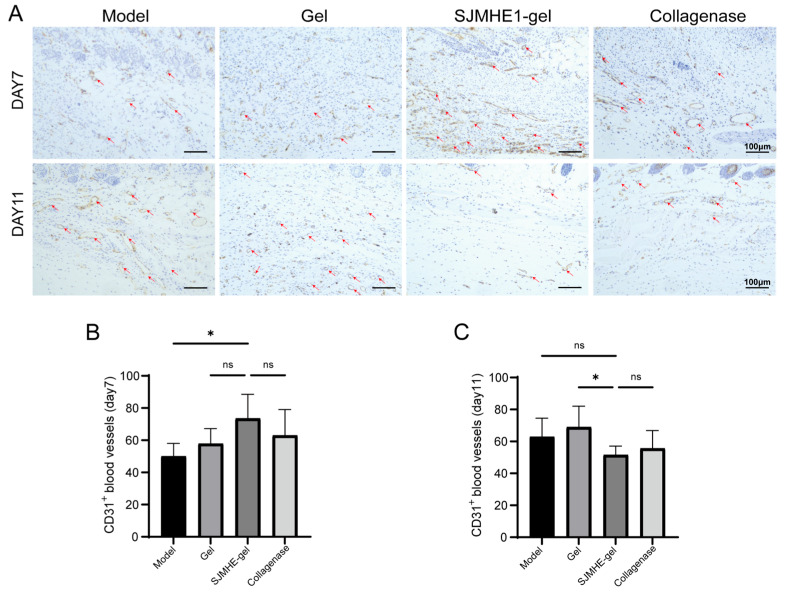
SJMHE1-gel enhances angiogenesis in early wound healing. (**A**) Representative images of immunohistochemical staining of CD31 on days 7 and 11. The red triangles indicate the CD31-positive expression of neovessels (scale bar = 100 μm). (**B**,**C**) Number of neovessels at the wound site on days 7 and 11 across groups. Data are presented as mean ± SD (*n* = 6). ns = not significant, * *p* < 0.05.

Neovascularisation is essential for supplying nutrients and oxygen to the wound during the proliferative phase, playing a critical role in early wound healing [38]. CD31, a marker for vascular endothelial cells, is commonly used to assess angiogenesis [39]. To evaluate the effect of the hydrogel on neovascularisation, CD31 immunohistochemical staining was performed. Figure 4A presents representative images of CD31 staining in wound tissues from each group of mice on days 7 and 11 post-wounding. On day 7, the number of CD31-positive microvessels in the wounds treated with SJMHE1-gel (73.5 ± 15.1 vessels/HPF) was significantly higher than in the model group (50.0 ± 8.0 vessels/HPF) (Figure 4B). However, by day 11, the density of CD31-positive microvessels in the SJMHE1-gel group (51.5 ± 5.6 vessels/HPF) had decreased significantly compared to the gel group (68.9 ± 13.1 vessels/HPF) (Figure 4C). Notably, within the SJMHE1-gel group, the density of CD31-positive microvessels exhibited a significant decrease from day 7 to day 11. In contrast, both the model group and the gel group showed an increase in microvessel number during this period. This suggests that SJMHE1-gel treatment accelerates the transition of wound healing from the proliferative phase to the remodelling phase.

### 3.5. SJMHE1-Gel Treatment Promotes M2 Macrophage Expression and Inhibits Inflammatory Responses

**Figure 5:**
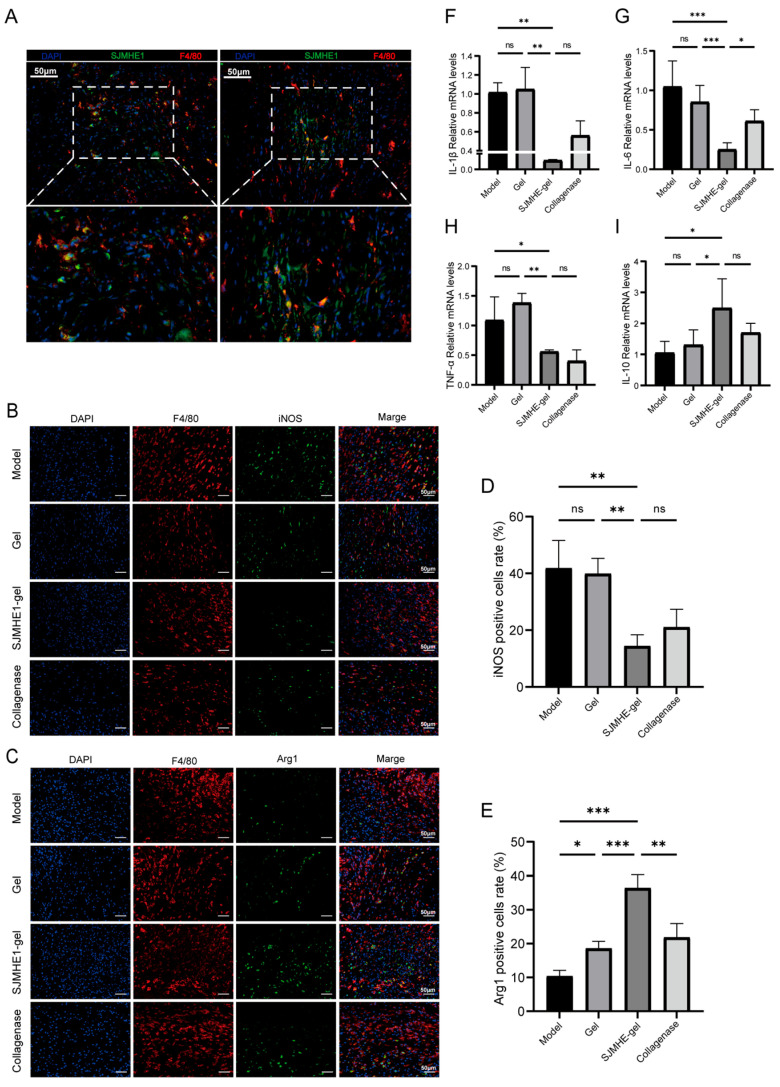
SJMHE1-gel treatment enhances M2 macrophage expression and suppresses inflammatory responses during wound healing in mice. (**A**) Representative immunofluorescence images showing SJMHE1 uptake by macrophages (scale bar = 50 μm). (**B**) Immunofluorescence images of iNOS, Arg1 (**C**), and F4/80 expression in wound tissue on day 7 in each group of mice (scale bar = 50 μm). (**D**) Quantitative analysis of iNOS, Arg1 (**E**), and F4/80 expression (*n* = 3). (**F**) Relative mRNA expression levels of IL-1β, IL-6 (**G**), TNF-α (**H**), and IL-10 (**I**) at the wound site in each group (*n* = 6). Data are presented as mean ± SD. ns = no significance, * *p* < 0.05, ** *p* < 0.01, and *** *p* < 0.001.

Macrophages are pivotal in wound healing [40]. To investigate whether the effects of SJMHE1-gel treatment on wound healing in mice are macrophage-mediated, mice were treated with FITC-labelled SJMHE1 hydrogel. The FITC-labelled SJMHE1 peptide (green) was co-localised with the macrophage marker F4/80 (red), as indicated by the yellow merged signal (Figure 5A).

Immunofluorescence staining on day 7 (Figure 5B–E) revealed that wounds treated with SJMHE1-gel exhibited a reduced number of pro-inflammatory iNOS-positive macrophages (14.4 ± 3.9%) and an increased expression of Arg1-positive macrophages (36.5 ± 3.9%) compared to the other groups. These results suggest that SJMHE1-gel treatment promotes M2 macrophage polarisation during skin wound healing.

In line with this increased M2 macrophage polarization, qPCR analysis (Figure 5F–I) demonstrated a significant reduction in mRNA expression levels of pro-inflammatory cytokines IL-1β, IL-6, and TNF-α, and a significant increase in the mRNA level of the anti-inflammatory cytokine IL-10 in the wound tissues of mice treated with SJMHE1-gel. These results indicate that SJMHE1-gel treatment inhibits wound inflammation by promoting M2 macrophage polarization in mice.

### 3.6. SJMHE1 Treatment Promotes M2 Macrophage-Related Gene Expression and Inhibits LPS-Induced Inflammatory Factor Expression In Vitro

**Figure 6:**
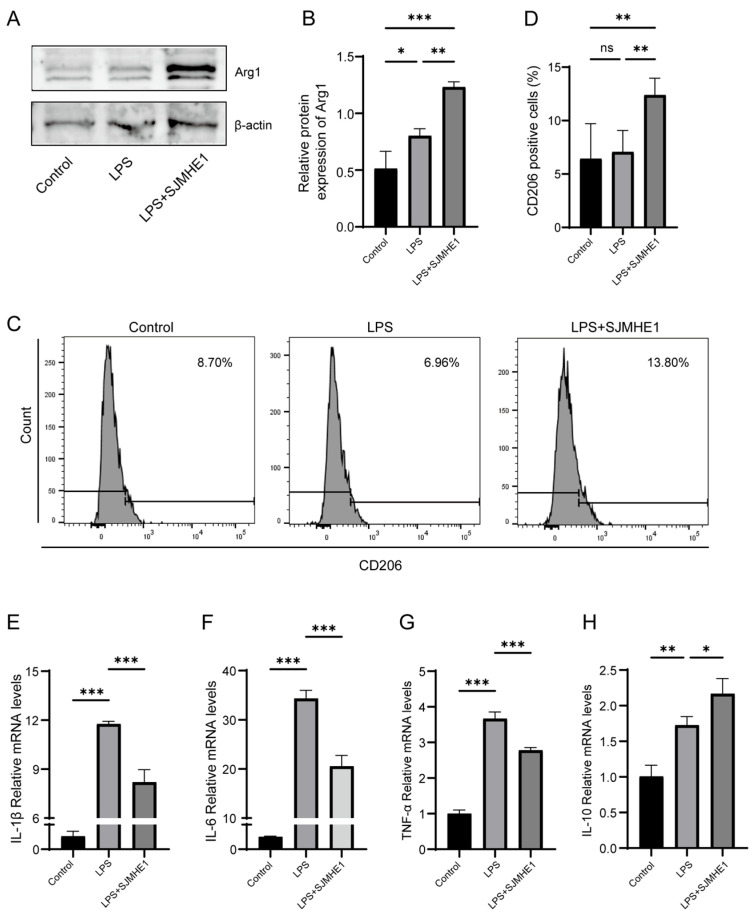
SJMHE1 treatment enhances M2 macrophage-related gene expression and reduces LPS-induced inflammatory factor expression in vitro. (**A**) Arg1 protein expression in RAW264.7 cells assessed by Western blotting. (**B**) Quantitative analysis of Arg1 protein expression. (**C**) Representative FACS plots of CD206. (**D**) Percentage of CD206+ cells in each group. (**E**) Relative mRNA expression levels of IL-1β, IL-6 (**F**), TNF-α (**G**), and IL-10 (**H**) in RAW264.7 cells. Data are presented as mean ± SD (*n* = 3). ns = no significance, * *p* < 0.05, ** *p* < 0.01, and *** *p* < 0.001. Original Western blot images are provided in the Supplementary Materials.

In vitro, SJMHE1 induction of M2 macrophages was assessed by evaluating the LPS-stimulated RAW264.7 cell phenotype and inflammatory factor expression. As shown in Figure 6A,B, SJMHE1 pretreatment significantly enhanced Arg1 protein expression compared to LPS stimulation alone, as determined by Western blot analysis. SJMHE1 pretreatment also increased the proportion of CD206-positive cells, as demonstrated by flow cytometry (Figure 6C,D). Furthermore, SJMHE1 pretreatment attenuated the LPS-induced mRNA expression of pro-inflammatory cytokines IL-1β, IL-6, and TNF-α while promoting the mRNA expression of the anti-inflammatory cytokine IL-10 (Figure 6E–H). These results suggest that SJMHE1 promotes M2 macrophage polarisation and inhibits the expression of inflammatory factors in vitro.

### 3.7. SJMHE1-Induced Macrophages Enhance Fibroblast and HUVEC Migration via TGF-β1/Smad3 Pathway, and Promote HUVEC Tubulogenesis Through VEGFA Upregulation

**Figure 7:**
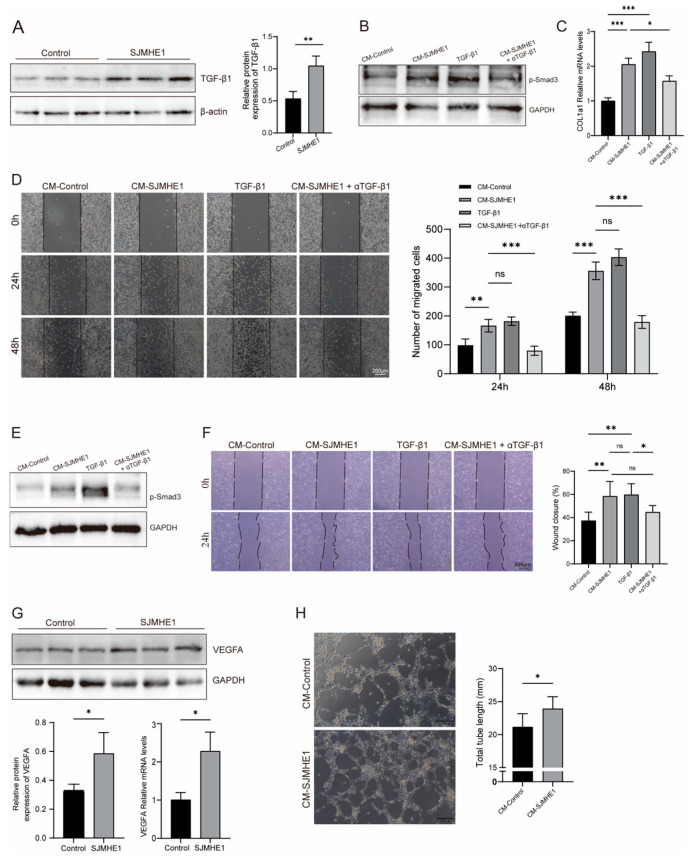
Macrophages treated with SJMHE1 promote fibroblast and HUVEC migration through TGF-β1/Smad3 pathway and VEGFA expression. (**A**) TGF-β1 protein expression in RAW264.7 cells assessed by Western blotting. (**B**) p-Smad3 protein expression in L929cells assessed by Western blotting. (**C**) Relative mRNA expression of COL1a1 in L929 cells. (**D**) Cell scratch assay and number of migrating L929 cells (scale bar = 200 μm). (**E**) p-Smad3 protein expression in HUVECs assessed by Western blotting. (**F**) Cell scratch assay and migration of HUVECs (scale bar = 200 μm). (**G**) VEGFA expression in THP-1-induced macrophages. (**H**) Representative images of vascular network formation and tube length quantification in HUVECs in vitro (scale bar = 200 μm). Data are presented as mean ± SD (*n* = 3). ns = no significance, * *p* < 0.05, ** *p* < 0.01, and *** *p* < 0.001. Original Western blot images are provided in the Supplementary Materials.

M2 macrophages release growth factors such as TGF-β and vascular endothelial growth factor (VEGF), which facilitate the transition to the proliferative phase of wound healing [41,42]. The previous results demonstrated that SJMHE1 stimulated the expression of M2 macrophages both in vitro and in vivo. Consequently, the expression of TGF-β1 in SJMHE1-treated macrophages was further examined. We found that SJMHE1 treatment significantly upregulated TGF-β1 expression in macrophages (Figure 7A). This increase in TGF-β1 further activated the TGF-β1/Smad3 signaling pathway in L929 fibroblasts and HUVECs, as evidenced by elevated levels of phosphorylated Smad3 (p-Smad3) (Figure 7B,E). Scratch assays revealed that conditioned medium from SJMHE1-treated macrophages (CM-SJMHE1) markedly accelerated the migration of L929 fibroblasts and HUVECs. The promigratory effect of CM-SJMHE1 was significantly attenuated in fibroblasts by a TGF-β1 neutralizing antibody (5 μg/mL) and partially inhibited in HUVECs (Figure 7D,F). Moreover, both CM-SJMHE1 and recombinant TGF-β1 upregulated COL1a1 mRNA expression in fibroblasts, and antibody blockade confirmed that this upregulation was TGF-β1-dependent (Figure 7C). SJMHE1 also significantly increased VEGFA expression in macrophages (Figure 7G). CM-SJMHE1 induced a denser and more branched tube-like network structure in HUVECs in vitro, with a total tube length significantly greater than that of the control group (Figure 7H). Collectively, these results indicate that SJMHE1 promotes the expression of TGF-β1 in macrophages, activating the TGF-β1/Smad3 pathway, thereby enhancing cell migration and collagen deposition, while concomitantly promoting HUVEC tubulogenesis through upregulation of VEGFA expression.

### 3.8. SJMHE1-Gel Treatment Activates the TGF-β1/Smad3 Signaling Pathway and Upregulates VEGFA in Wound Tissue of Mice

**Figure 8:**
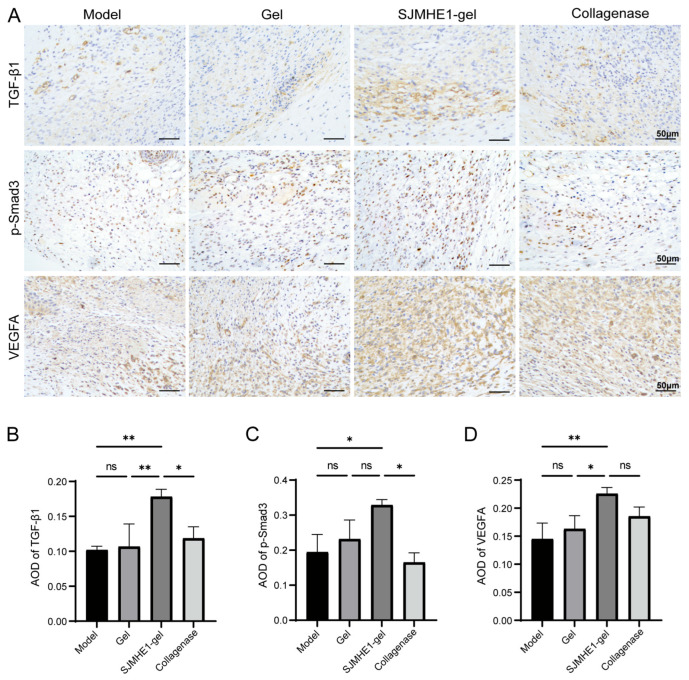
SJMHE1-gel treatment increases TGF-β1, p-Smad3, and VEGFA expression in mice. (**A**) Representative immunohistochemical staining images of TGF-β1, p-Smad3, and VEGFA on day 7 (scale bar = 50 μm). (**B**) Quantification of TGF-β1, p-Smad3 (**C**) and VEGFA (**D**) expression. Data are presented as mean ± SD (*n* = 6). ns = no significance, * *p* < 0.05, ** *p* < 0.01.

To further explore the effects of SJMHE1 on growth factors in vivo, the expression of TGF-β1 and VEGFA in wound tissue from mice was assessed. Given the transition of macrophages from the inflammatory to the proliferative phase, wound tissues from day 7 were selected to detect TGF-β1, p-Smad3 and VEGFA expression (Figure 8A). Consistent with the in vitro results, SJMHE1-gel-treated mice exhibited a significant increase in TGF-β1, p-Smad3 and VEGFA expression compared to the control groups (Figure 8B–D). Additionally, the results showed that p-Smad3 expression was exhibited nuclear enrichment in the SJMHE1-gel group (Figure 8A), indicating that the TGF-β1/Smad3 signaling pathway was activated. These results indicate that SJMHE1-gel treatment effectively activates the TGF-β1/Smad3 signaling pathway in wound tissue while simultaneously upregulating VEGFA expression, promoting epithelial regeneration, collagen deposition, and angiogenesis at the wound site.

## 4. Discussion

The incidence and frequency of chronic wounds are increasing due to an ageing population, leading to a continuous rise in social and economic burdens. Traditional wound dressings, topical medications, and surgery are often insufficient in restoring normal skin anatomy and structure [43]. Novel approaches to wound healing, such as stem cell therapies and 3D bioprinting, are not only costly but also require specialised technical expertise [44]. Additionally, stem cell therapy must face challenges related to tumorigenicity and immune rejection [45], highlighting the need for simple yet effective alternatives. This study demonstrates that SJMHE1-encapsulated glycogen hydrogel effectively remodels the wound microenvironment by inducing M2 macrophages, reducing inflammation, and promoting angiogenesis and shows promise for acute wound healing.

Hydrogels protect encapsulated protein-based drugs from rapid hydrolysis by proteases, thereby controlling drug release [46]. The release profile of glycogen hydrogel-loaded peptides demonstrates that SJMHE1 release is effectively delayed. Similar to mesenchymal stem cell compatibility [47], glycogen-loaded SJMHE1 hydrogels are non-toxic to L929 cells and do not cause hemolysis. Additionally, this hydrogel promotes skin wound healing in mice.

The wound healing process involves a complex series of phases—hemostatic, inflammatory, proliferative, and remodelling—each occurring in a specific sequence, with overlapping stages. Our findings indicate that glycogen-loaded SJMHE1 peptide hydrogel accelerates the early stages of wound healing, promoting granulation tissue formation and angiogenesis. This leads to faster progression into the remodelling phase, characterised by increased collagen deposition and elevated expression of collagen type I in the later stages. Moreover, the hydrogel promotes the regeneration of skin follicles and accessory glands at the wound site. These results suggest that the hydrogel enhances the structural normalisation and epithelial regeneration of injured skin, facilitating rapid wound healing in mice.

The phenotypic shift of macrophages during inflammation is a crucial step in wound healing. M1 macrophages, present during acute injury, transition to the M2 phenotype during the repair phase, which plays a key role in anti-inflammatory responses and tissue regeneration [40,48]. FITC-SJMHE1-gels were successfully captured by macrophages at the site of skin injury (Figure 5A). In line with SJMHE1′s promotion of M2 macrophage polarisation in peripheral nerve repair [23], SJMHE1-encapsulated glycogen hydrogel stimulates M2 marker Arg1 expression, suppresses M1 marker iNOS expression, reduces IL-1β, IL-6, and TNF-α mRNA expression, and enhances IL-10 mRNA expression at the site of skin injury. Similarly, in vitro, SJMHE1-treated RAW264.7 macrophages exhibited high expression of the M2 markers Arg1 and CD206, inhibited LPS-stimulated IL-1β, IL-6, and TNF-α mRNA expression, and promoted IL-10 mRNA expression.

M2 macrophages secrete growth factors such as VEGFA, PDGF, and TGF-β1, which promote angiogenesis, epithelial regeneration, and collagen production, facilitating the transition to the proliferative phase of wound healing [49]. During this phase, fibroblasts are recruited to the wound site and begin producing key components of the extracellular matrix, including collagen, proteoglycans, fibronectin, and elastin [50,51]. These fibroblasts undergo proliferation, migration, and differentiation, driven by growth factors like PDGF and TGF-β. Fibroblasts and endothelial cells are essential for capillary growth and granulation tissue formation at the site of dermal wound healing [42,50].

TGF-β1, a multifunctional cytokine [52,53], plays a critical role throughout the wound healing process by modulating inflammation, promoting granulation tissue formation, and facilitating the migration of fibroblasts and myofibroblasts to the injury site [42,54]. Our study observed increased TGF-β1 and p-Smad3 expression in SJMHE1-treated macrophages. Both SJMHE1-treated macrophage-CM and recombinant TGF-β1 promoted fibroblast and HUVEC migration. However, the addition of TGF-β1-neutralising antibodies to the CM significantly attenuated fibroblast and HUVEC migration, indicating that SJMHE1-treated macrophages enhance fibroblast and HUVEC migration via TGF-β1/Smad3 pathway. Activated TGF-β1 induces the transcription of collagenase genes, marking the progression to the remodelling phase of wound healing [55]. Moreover, CM from SJMHE1-treated macrophages stimulated fibroblasts to express COL1a1 mRNA at high levels, a finding consistent with the elevated COL1a1 expression observed in mouse wound tissues following treatment with SJMHE1-encapsulated glycogen hydrogel. These results suggest that SJMHE1 accelerates wound remodelling both in vitro and in vivo.

Angiogenesis is a critical stage in wound healing [56,57,58]. VEGFA released by macrophages promotes collagen deposition, angiogenesis, and skin regeneration. Fibroblasts and vascular endothelial cells migrate to the wound site to form granulation tissue and neovascularisation, ensuring an adequate supply of nutrients for the healing phase and supporting the growth of newly formed tissue [55,56,57]. CM from SJMHE1-treated macrophages also enhances VEGFA expression, as well as HUVEC migration and tube formation. Moreover, the high VEGFA expression in SJMHE1-treated macrophages was consistent with the elevated vascular marker CD31 at the wound site in mice treated with glycogen-loaded SJMHE1 hydrogel, indicating that SJMHE1 accelerates angiogenesis both in vitro and in vivo, thereby facilitating wound remodelling and healing. In line with the in vitro findings, glycogen-loaded SJMHE1 hydrogel-treated mice also exhibited increased TGF-β1, p-Smad3 and VEGFA expression at the wound sites. These results collectively demonstrate that SJMHE1 exerts multifunctional therapeutic effects by inducing M2 macrophages, alleviating inflammation, and accelerating wound remodelling and healing through the TGF-β1/Smad3 signaling pathway and upregulation of VEGFA expression. However, this study only evaluated the efficacy of SJMHE1-gel in excisional wound healing in mice, and thus has certain limitations. Further research is needed to determine whether the SJMHE1 natural biomaterial hydrogel can be applied to wound healing in humans or other species. Furthermore, given its multiple favorable properties, the potential of SJMHE1 hydrogel for treating complex wounds—such as full-thickness skin burns or pressure ulcers—also warrants in-depth exploration.

## 5. Conclusions

In summary, a new, simple, SJMHE1-encapsulated glycogen hydrogel was developed to promote acute wound healing. This hydrogel fosters angiogenesis and collagen deposition, reduces inflammation, and enhances the re-epithelialization of wounds in mice. SJMHE1 mechanistically reduces inflammation while accelerating wound remodeling and healing by inducing M2 macrophages, upregulating TGF-β1 and VEGFA expression, and promoting the migration of fibroblasts and vascular endothelial cells. Compared to the positive control group using collagenase ointment, the glycogen-loaded SJMHE1 hydrogel demonstrated faster wound healing and a higher healing rate during the early stages (0–7 days). This cost-effective, easy-to-prepare, safe hydrogel holds significant potential for application and offers a promising new approach for skin wound healing.

## Data Availability

The original contributions presented in this study are included in the article/Supplementary Materials. Further inquiries can be directed to the corresponding author(s).

## Supplementary Materials

The following supporting information can be downloaded at: https://www.mdpi.com/article/10.3390/biom16030392/s1, Figure S1: Photograph of the SJMHE1-gel placed in an inverted/tilted position; File S1: Original Western blot images for Figure 6 and Figure 7.
