# Supplementary figures and images for: Glycogen Hydrogel Loaded with Schistosoma japonicas Peptide SJMHE1 Improves Skin Wound Healing
Source: Biomolecules. 2026 Mar 5;16(3):392. doi: 10.3390/biom16030392 (PMC13024005; doi:10.3390/biom16030392)

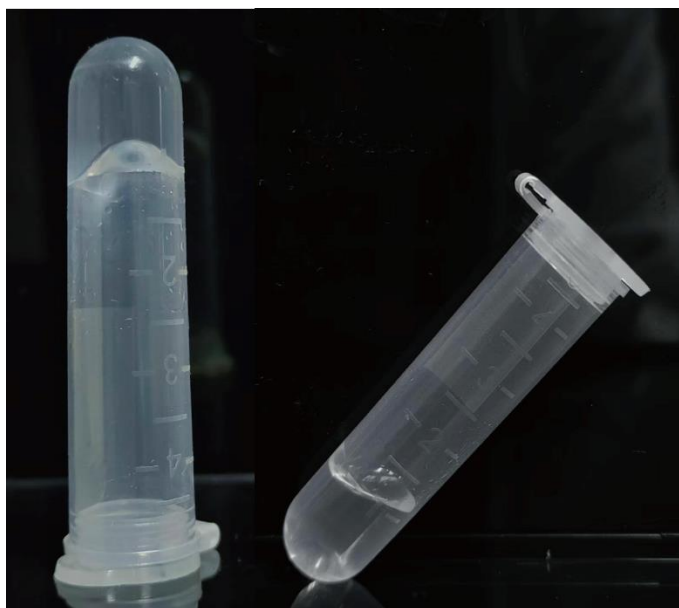

Figure S1. Photograph of the SJMHE1-gel placed in an inverted/tilted position.

Supplement: Supplementary file 1 [file biomolecules-16-00392-s001.zip › Figure S1.pdf]

**Fig 6A**

70kDa  
55kDa  
40kDa  
35kDa

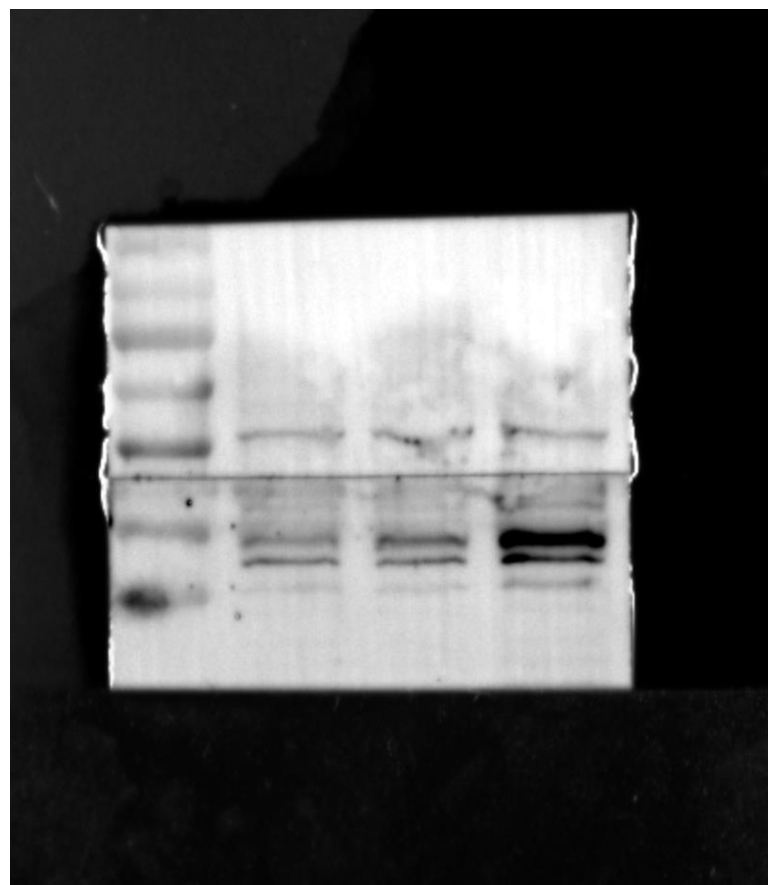

Control  
LPS  
LPS+SJMHE1

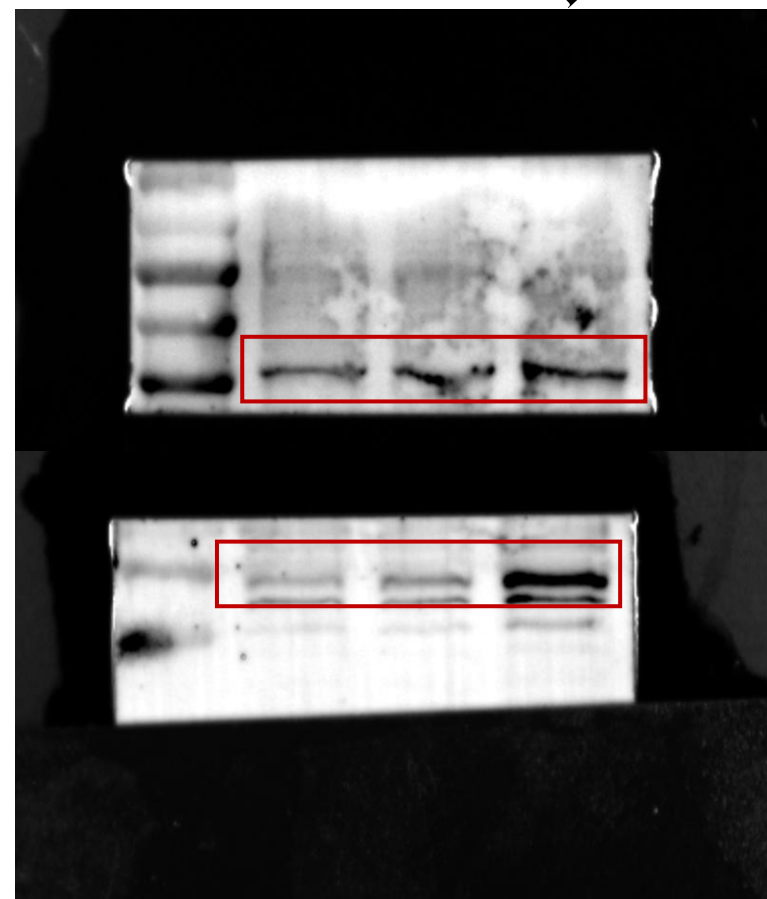

←  $\beta$ -actin (43kDa)

← Arg1 (34kDa)

**Fig 7A**

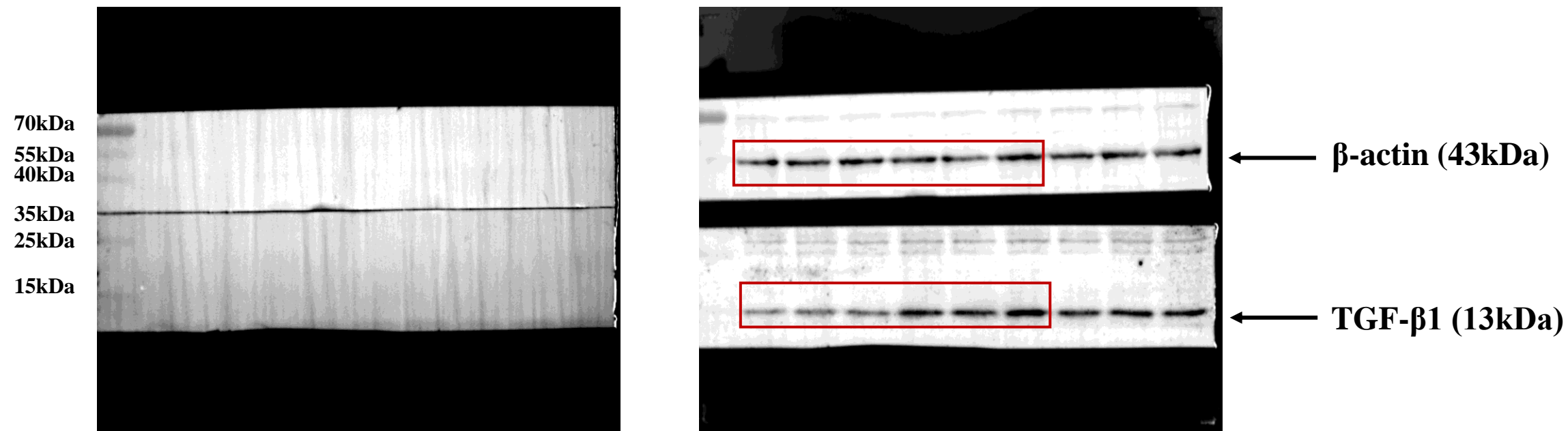

**Fig 7B**

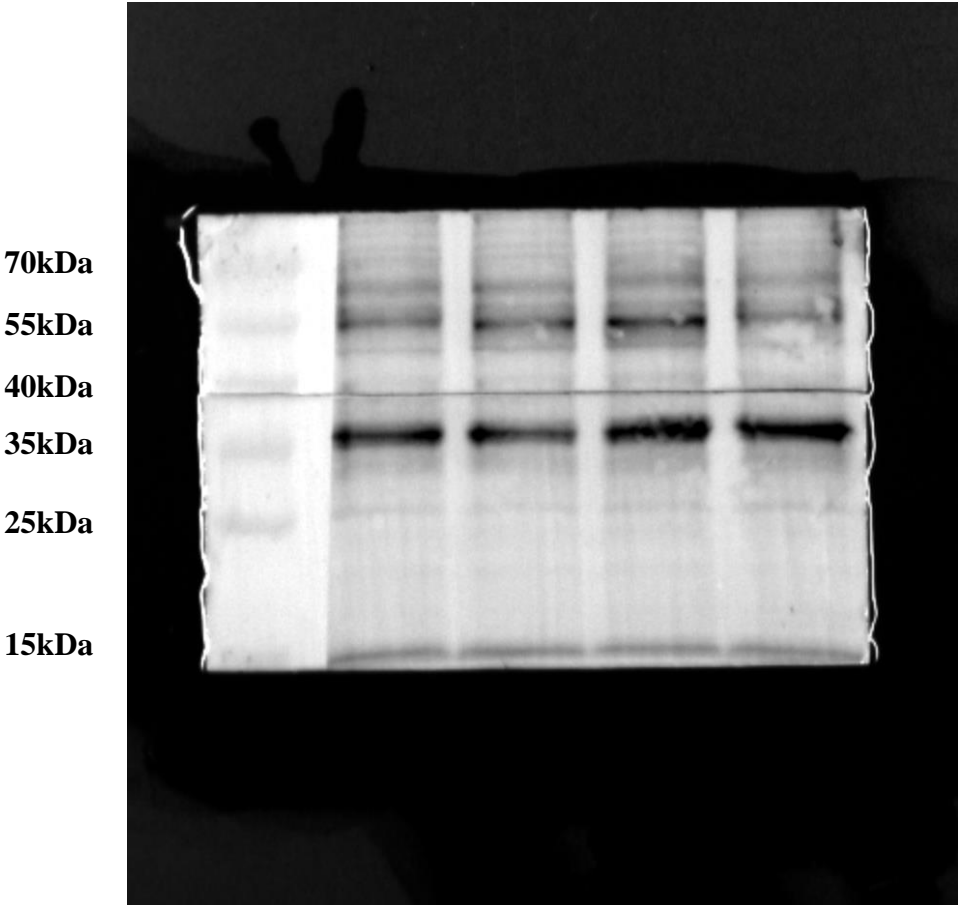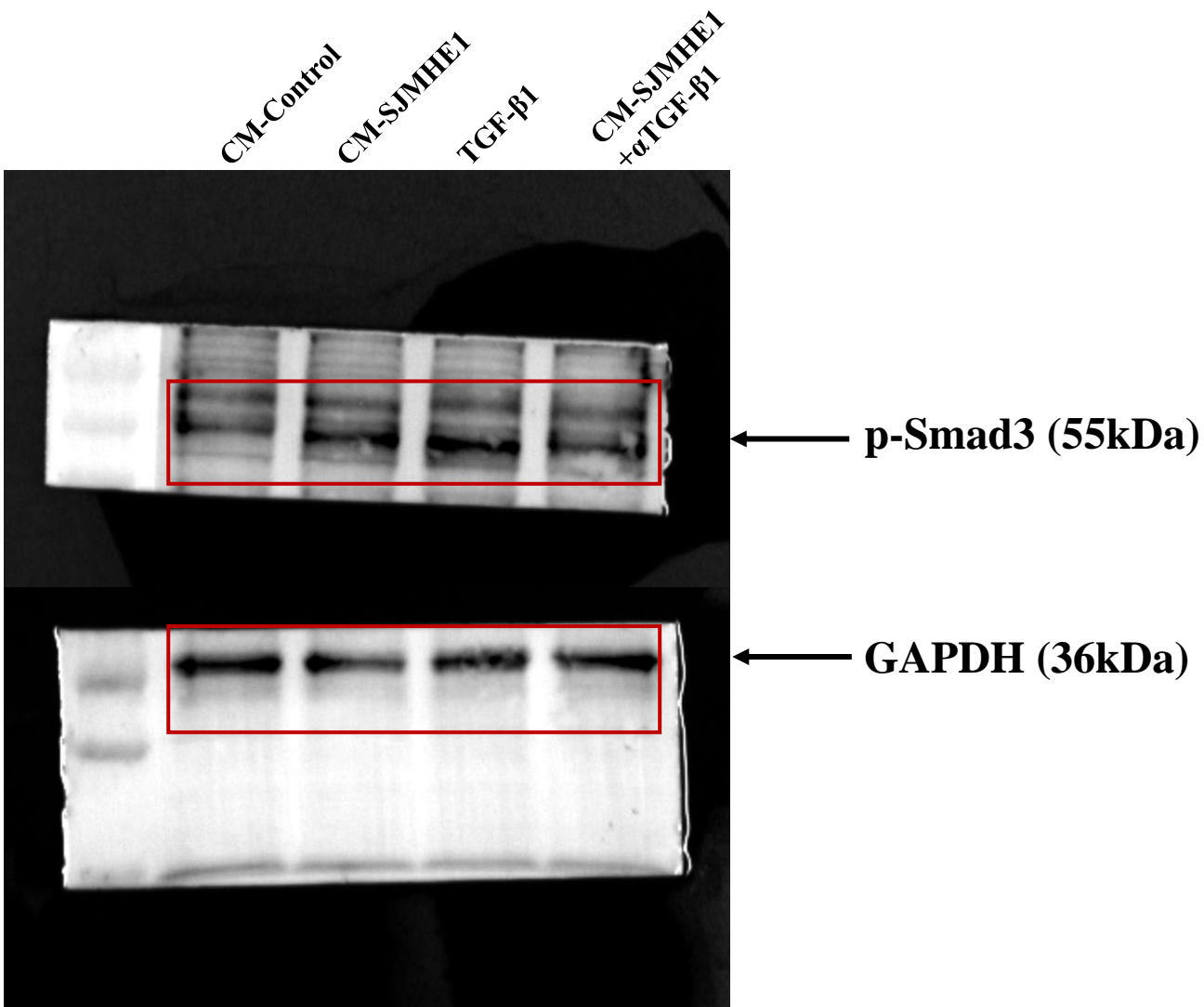

**Fig 7E**

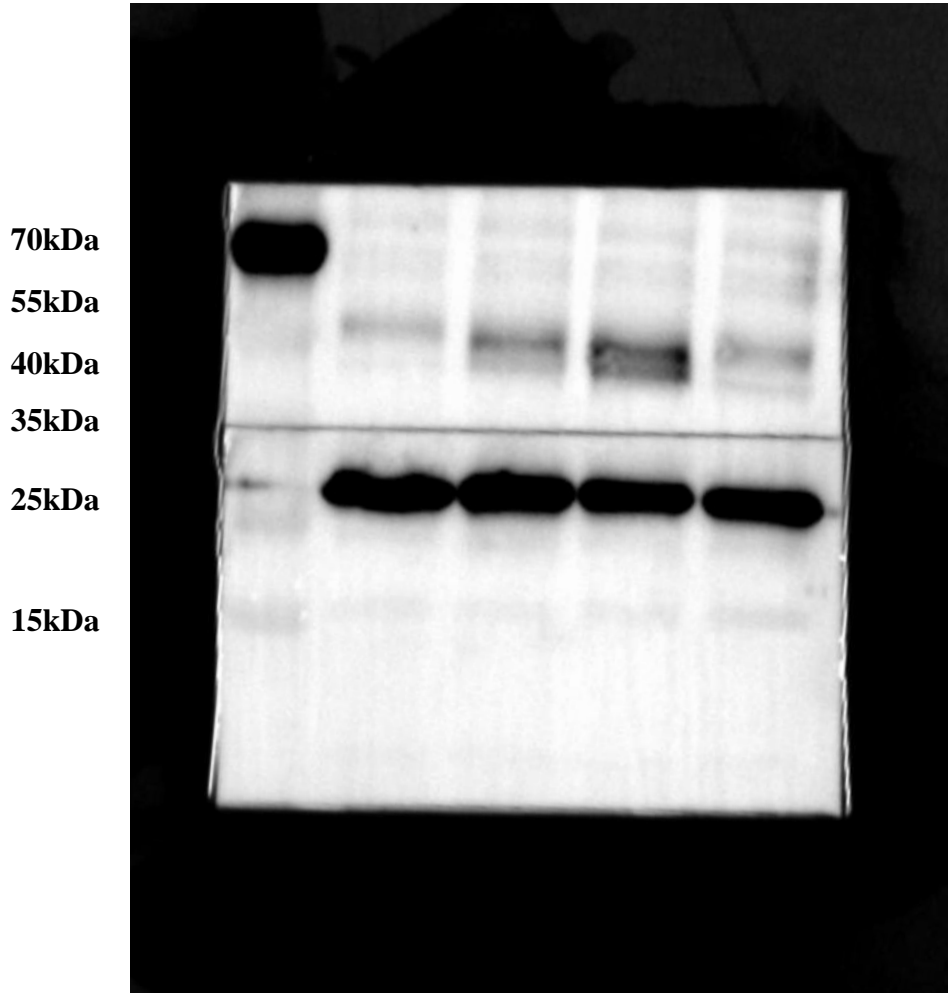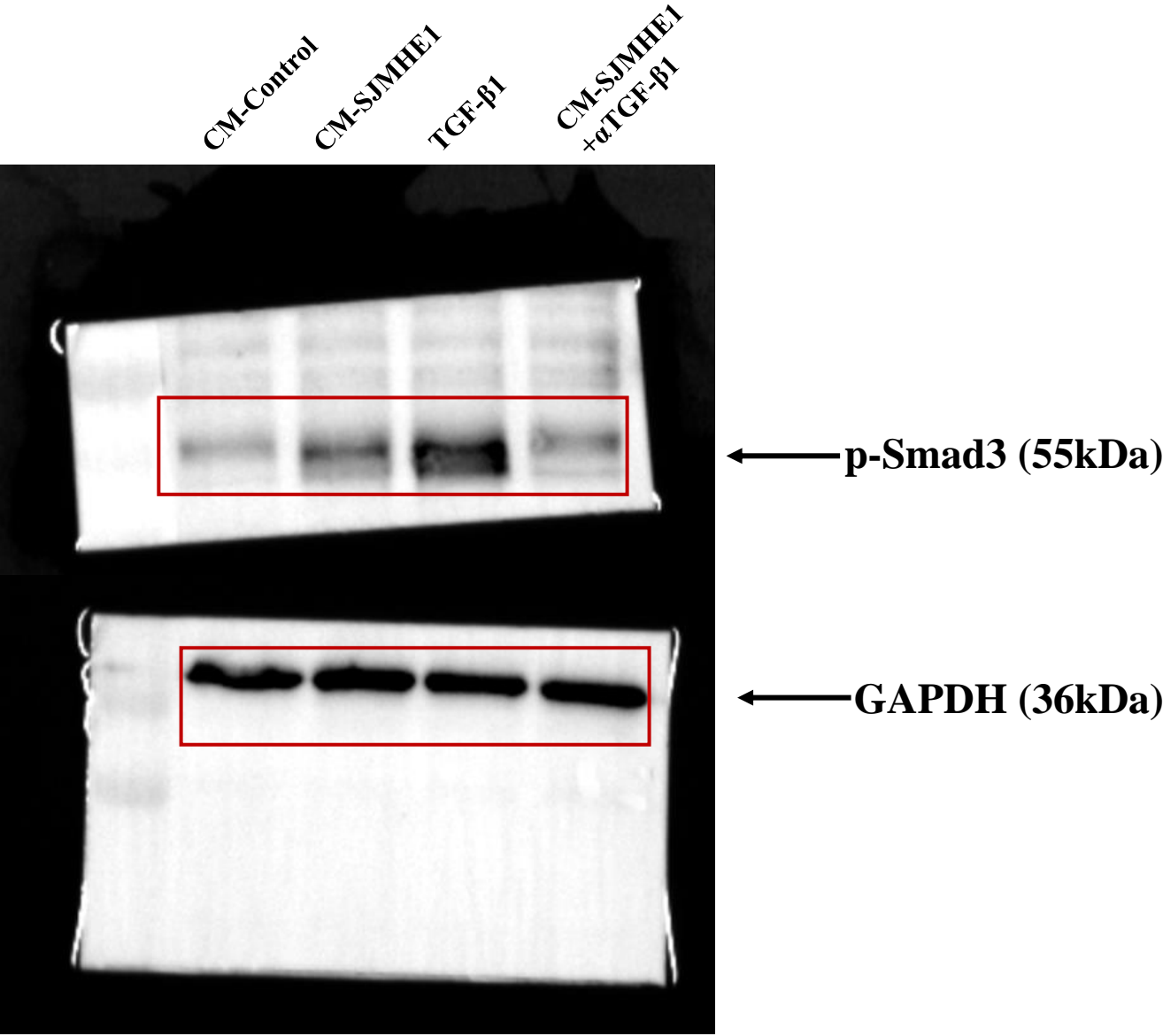

**Fig 7G**

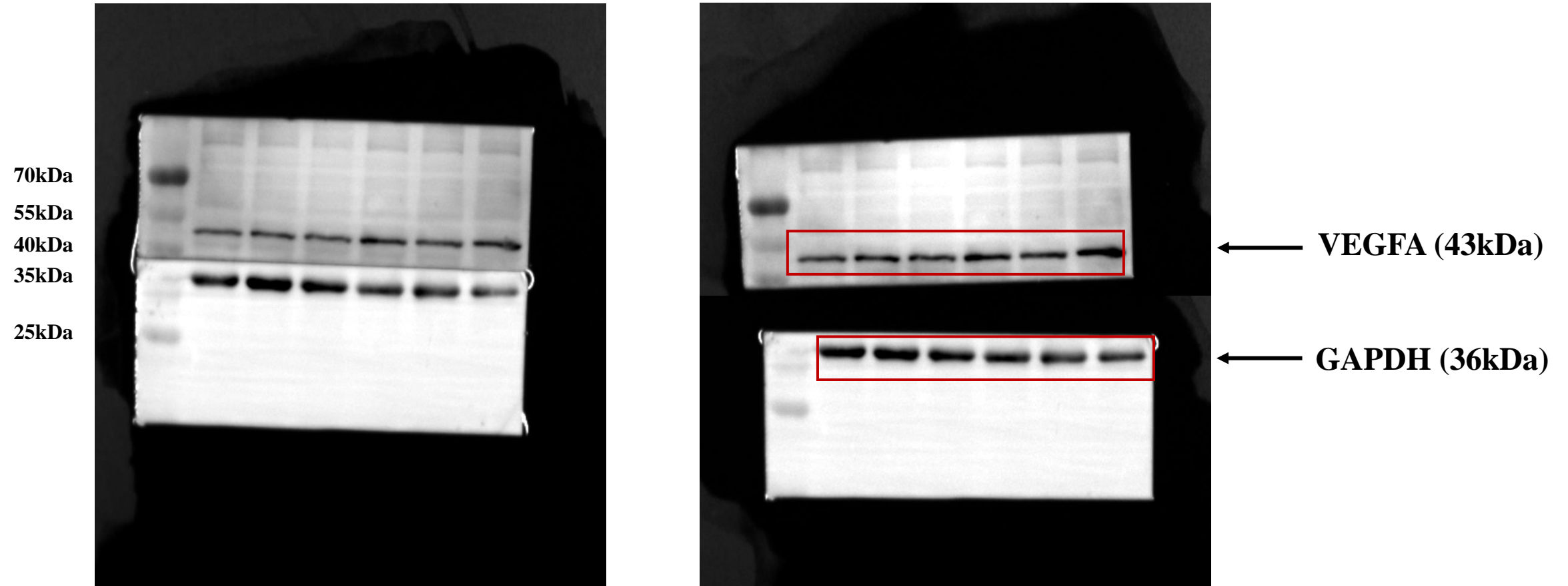

Supplement: Supplementary file 1 [file biomolecules-16-00392-s001.zip › Original Western blot images for Figure 6 and Figure 7.pdf]
